# From intestinal barrier sensing to glial immune programming: a barrier–immune–glia relay in microbiota-associated neurodegeneration

**DOI:** 10.3389/fimmu.2026.1956619

**Published:** 2026-10-27

**Authors:** Juanna Lin, Xuan Zou

**Affiliations:** 1 Department of Pharmacy, The First Affiliated Hospital of Shantou University Medical College, Shantou, China; 2 Department of Pain Medicine, Beijing University of Chinese Medicine Affiliated Shenzhen Hospital (Longgang), Shenzhen, China

**Keywords:** astrocytes, blood-brain barrier, gut microbiota, gut-immune-brain axis, intestinal mucosal barrier, microglia, neurodegenerative diseases, neuroimmunology

## Abstract

The gut microbiota contributes to host homeostasis through metabolic, immunological, neural, and barrier-related pathways, and its role in the onset and progression of neurodegenerative diseases has received increasing attention. Patients with Alzheimer’s disease, Parkinson’s disease, and amyotrophic lateral sclerosis often exhibit alterations in gut microbial composition, microbial metabolic function, and peripheral inflammatory phenotypes. However, existing clinical evidence is derived predominantly from cross-sectional studies, and the direction of causality between microbiota alterations and disease pathology remains to be established. This review uses a “barrier-immune-glial relay” framework to systematically summarize the potential mechanisms through which gut microbiota-associated signals influence immune homeostasis in the central nervous system. First, alterations in microbial composition, short-chain fatty acids, bile acids, tryptophan derivatives, and lipopolysaccharide may act on the intestinal epithelial barrier, enteric nervous system, and intestinal mucosal immune cells, thereby affecting local immune tolerance and inflammatory responses. Second, intestinal macrophages, dendritic cells, innate lymphoid cells, T cells, and B cells may translate local microbial signals into circulating metabolites, inflammatory mediators, and immune cell-associated signals, which may in turn influence blood-brain barrier function and the central immune microenvironment. Furthermore, microglia and astrocytes, as key integrators within the central nervous system, can respond to gut-derived metabolites and peripheral immune signals. Their interaction network may determine whether neuroinflammation, synaptic injury, and neurodegenerative pathology are amplified or constrained. This review also evaluates the levels of evidence supporting these mechanisms in Parkinson’s disease, Alzheimer’s disease, amyotrophic lateral sclerosis, and multiple sclerosis. Although these disorders may share mechanistic nodes, including barrier impairment, immunometabolic dysregulation, and glial responses, their core pathologies, immune contexts, and temporal disease trajectories differ substantially. Finally, we propose that establishing a verifiable causal chain linking “microbial functional genes-metabolites-barrier and immune remodeling-glial cell states-neuropathology-clinical phenotypes” will provide a basis for biomarker development and precision interventions in neurodegenerative diseases.

## 1 Introduction

The gut microbiota is an important ecological and immunoregulatory system of the host. Its functions extend beyond nutrient utilization, energy metabolism, and the maintenance of local intestinal homeostasis, and include the regulation of distant organ functions through the enteric nervous system (1), vagus nerve (2), neuroendocrine pathways (3), circulating metabolites (4), and immune signals (5). In recent years, studies involving germ-free animals, antibiotic interventions, and microbial colonization have demonstrated that the host microbiota is closely associated with central nervous system (CNS) development and immune homeostasis (6). The gut microbiota can influence blood-brain barrier (BBB) integrity, microglial maturation and function, neurogenesis, and neuroinflammatory responses (7). These findings have shifted gut-brain axis research from conventional neuroendocrine and behavioral observations toward mechanistic investigations centered on immune regulation, barrier function, and intercellular communication.

Neurodegenerative diseases, including Alzheimer’s disease (AD), Parkinson’s disease (PD), and amyotrophic lateral sclerosis (ALS), commonly share features such as chronic neuroinflammation (8), protein misfolding (9), synaptic injury (10), neuronal loss (11), and aberrant glial responses (12). An increasing number of clinical cohort studies have identified alterations in gut microbial structure, microbial metabolic function, and fecal or circulating metabolite profiles in patients with PD and AD (13, 14). Taxa repeatedly discussed across these disorders include *Akkermansia muciniphila* (15), *Faecalibacterium prausnitzii* (16), *Roseburia* spp (17)., *Bifidobacterium* spp (18)., *Lactobacillus* spp (19)., *Enterobacteriaceae* (20), and *Desulfovibrio spp* (21). However, the biological relevance of these taxa should not be inferred from changes in taxonomic abundance alone, but should be interpreted in conjunction with their functional capacity, ecological context, and metabolite output. In addition, patients with PD frequently present with constipation (22), intestinal inflammation (23), and gastrointestinal dysmotility (24), whereas patients with AD may also exhibit altered nutritional status (25), metabolic comorbidities (26), and exposure to multiple medications (27). These factors may both contribute to disease-associated alterations in the intestinal microenvironment and, conversely, reshape microbial composition. Therefore, the association between gut dysbiosis and neurodegenerative diseases cannot be directly equated with a unidirectional causal relationship. Its pathological significance must be clarified after rigorous control for confounding factors, including age, diet, medication use, disease stage, and host genetic background.

Current research characterizes the gut-brain axis as a multidirectional communication network that integrates neural, endocrine, immune, metabolic, and barrier-related pathways rather than a linear gut-to-brain route (28). Major microbiota-associated metabolites arise from both dietary substrates and host-derived molecules that are subsequently transformed by intestinal microbes. Short-chain fatty acids (SCFAs), including acetate, propionate, and butyrate, are primarily produced through the anaerobic fermentation of non-digestible dietary carbohydrates and fiber (29). Bile acids are synthesized in the liver as primary bile acids and are further modified by gut bacteria through deconjugation, dehydroxylation, and other biotransformation reactions to generate secondary bile acids (30). Dietary tryptophan can be metabolized by host cells and gut microbes into multiple bioactive derivatives, including indole compounds and kynurenine-related metabolites (31). In addition, lipid metabolites may originate from dietary lipids, host phospholipids, and microbial lipid metabolism, with subsequent modification by the gut microbiota (32). These microbiota-associated signals, together with microbe-associated molecular patterns such as lipopolysaccharide (LPS), can act on intestinal epithelial cells (33), the mucus layer (34, 35), tight-junction complexes (35), and intestinal mucosal immune cells (36). Through these interactions, gut-derived signals may regulate intestinal barrier integrity, immune tolerance, and local inflammation (37). They may subsequently influence the central nervous system (CNS) through several interconnected routes, including the enteric nervous system and vagus nerve, circulating microbial metabolites, peripheral cytokines, immune-cell trafficking, and endocrine signaling. Intestinal macrophages, dendritic cells, innate lymphoid cells, and T and B lymphocytes can integrate microbial and host-derived signals and shape systemic immune responses through cytokine production, metabolite release, and immune-cell-mediated communication (38). In parallel, the intestinal barrier, blood–brain barrier (BBB), and cerebrovascular immune microenvironment serve as important regulatory interfaces that control the exchange of metabolic, inflammatory, and immune signals between the periphery and the CNS (39). Within the CNS, microglia and astrocytes can sense and integrate peripherally derived metabolic and inflammatory signals, thereby influencing synaptic function (40), neuronal survival (41), abnormal protein clearance (41), and the progression of neuroinflammation (42). Rothhammer et al. demonstrated that microbiota-derived tryptophan metabolites can regulate astrocyte responses through aryl hydrocarbon receptor signaling (43), supporting a role for metabolite-sensitive glial pathways in the communication between the gut and CNS. More broadly, reciprocal interactions among microbial metabolites, peripheral immune cells, endothelial and barrier cells, microglia, and astrocytes may constitute important mechanistic nodes through which gut-derived signals influence central inflammation and neurological disease (44, 45). These findings underscore that the biological effects of the gut microbiota depend not only on microbial composition, but also on the nature, concentration, and bioavailability of microbial metabolites, as well as their effects on neural, endocrine, immune, metabolic, and barrier functions.

Despite these advances, several important limitations remain. First, many studies have focused primarily on taxonomic differences in the gut microbiota and have not adequately characterized the relationships among microbial functions, metabolite alterations, and host immune responses. Second, isolated observations—such as increased BBB permeability, peripheral inflammation, or glial activation—are insufficient to establish that gut-derived signals constitute a pathogenic causal chain across biological barriers. Third, although different disorders may share mechanistic nodes, including intestinal barrier impairment, immunometabolic dysregulation, BBB abnormalities, and altered glial responses, their disease-defining pathologies and immune contexts differ substantially. PD is primarily characterized by α-synuclein pathology, AD by amyloid-β (Aβ) and tau pathology, ALS by progressive motor-neuron degeneration in the context of disease-specific genetic and proteinopathic mechanisms, and MS by immune-mediated demyelination and neuroaxonal injury. Therefore, microbiota-related findings must be interpreted in a disease- and stage-specific manner.

Against this background, this review builds on evidence linking gut dysbiosis to neurodegenerative and neuroinflammatory diseases by using the “barrier–immune–glial relay” as a complementary framework. We examine how microbiota-derived signals are sensed by the intestinal barrier and mucosal immune system and how they may influence peripheral immunity, BBB function, and central glial responses. Rather than proposing a universal or linear pathogenic cascade, this framework highlights shared biological interfaces whose direction, timing, and disease relevance may differ across disorders. We first summarize the roles of microbial metabolites, microbe-associated molecular patterns, barrier dysfunction, immune remodeling, and glial responses, and then evaluate their disease-specific evidence, limitations, and translational implications in PD, AD, ALS, and MS. Particular emphasis is placed on establishing longitudinal and experimentally testable links among microbial functions, metabolites, barrier integrity, immune states, glial activation, disease-specific pathology, and clinical phenotypes. Such an approach may help move the field beyond taxonomic descriptions of dysbiosis toward disease- and stage-specific mechanistic models and precision therapeutic strategies.

## 2 “Barrier-immune-glial relay”: gut microbiota regulation of neuroimmunity

Alterations in microbial composition, metabolic activity, and microbe-associated molecular patterns can influence intestinal epithelial cells, the mucus layer, the enteric nervous system, and immune cells in the lamina propria (46). Microbial products such as lipopolysaccharide (LPS) may disrupt epithelial tight-junction organization and increase intestinal permeability (47). However, intestinal barrier dysfunction is not attributable solely to microbiota-related changes. Dietary emulsifiers and surfactants, chelating agents, non-steroidal anti-inflammatory drugs (NSAIDs), chemotherapeutic agents, antibiotics, alcohol, and other medications may also impair mucus integrity, epithelial viability, or tight-junction function (48, 49). These exposures may act independently of, or interact with, microbial and host-related factors to facilitate the translocation of microbial products and inflammatory mediators into the circulation (50). Accordingly, intestinal barrier dysfunction in neurodegenerative diseases should be regarded as a multifactorial process, and medication use, comorbidities, diet, and nutritional status should be considered when interpreting microbiota–barrier associations (**Figure 1A**).

**Figure 1:**
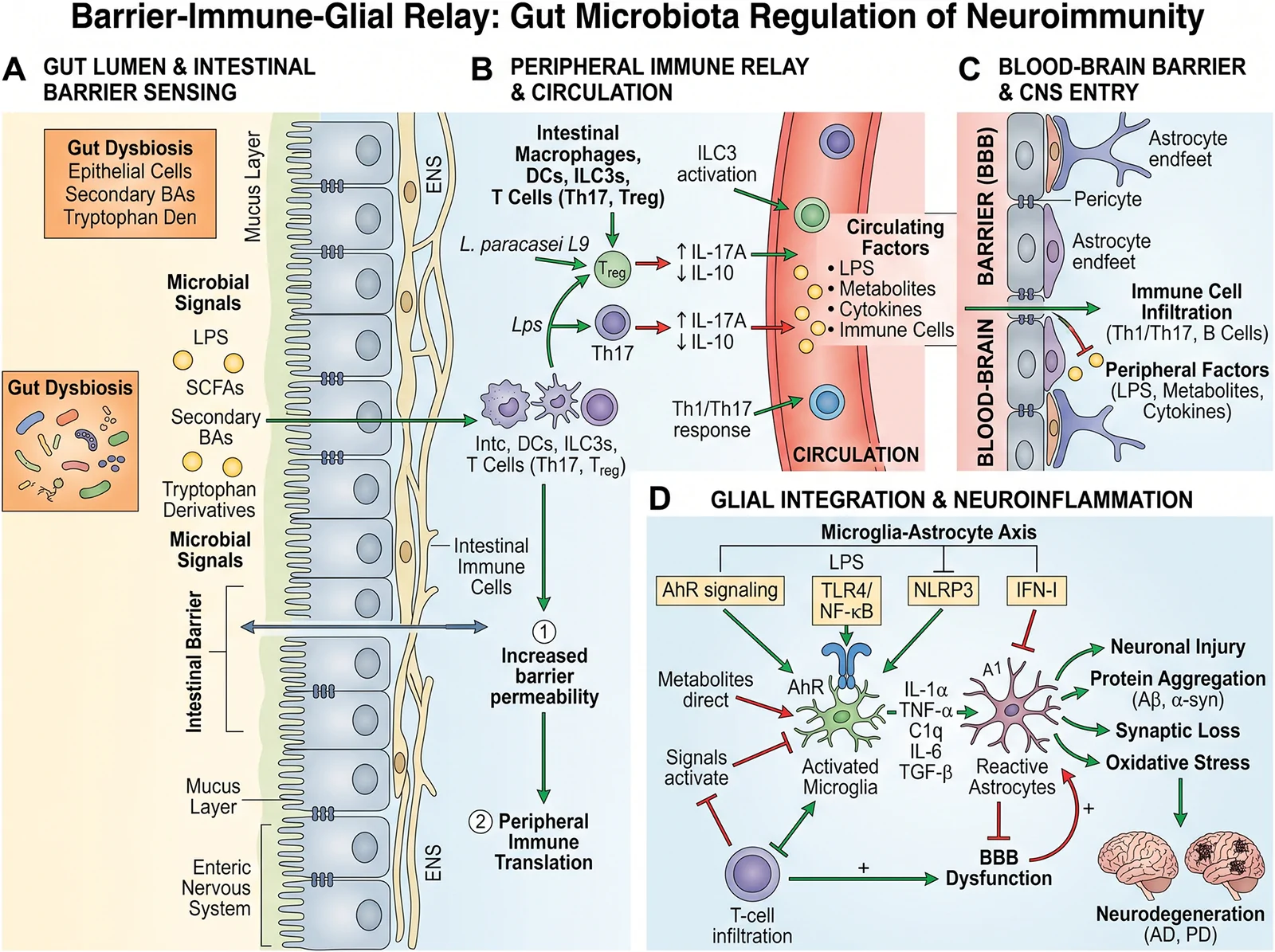
Schematic overview of how microbiota-associated intestinal barrier disruption promotes neurodegeneration through peripheral immune activation and glial reprogramming. **(A)** Gut microbial dysbiosis, microbial metabolites, pathogen-associated molecular patterns, and inflammatory stimuli can compromise intestinal epithelial barrier integrity, resulting in increased intestinal permeability. **(B)** Subsequent mucosal immune activation, release of pro-inflammatory cytokines, and recruitment of peripheral immune cells transmit inflammatory signals to the central nervous system through the circulation and may be accompanied by blood-brain barrier (BBB) dysfunction. **(C)** Inflammatory mediators and immune cells that enter or act on the central nervous system can further induce microglial and astrocytic activation, **(D)** thereby promoting the production of cytokines, reactive oxygen species, and other neurotoxic mediators. Persistent immune-glial crosstalk ultimately exacerbates synaptic injury, neuronal dysfunction, and neurodegenerative pathology. Green arrows indicate promoting or enhancing effects, whereas red arrows indicate injurious or pro-inflammatory effects. This figure summarizes putative mechanisms; the precise causal relationships and their relative contributions across neurodegenerative diseases require further validation.

Microbial signals are subsequently integrated by intestinal macrophages, dendritic cells, innate lymphoid cells, and T and B cells, which contribute to local immune tolerance or inflammation (51). Microbial metabolites can modulate cytokine networks and lymphocyte differentiation. For example, short-chain fatty acids, particularly butyrate, can promote Foxp3^+^ regulatory T-cell differentiation and enhance IL-10-associated anti-inflammatory signaling (52). Conversely, LPS can activate TLR4-dependent pathways in innate immune cells, inducing the production of TNF-α, IL-1β, and IL-6 and potentially enhancing downstream Th17-associated IL-17A responses (48). Tryptophan-derived indoles may further regulate mucosal immune responses through aryl hydrocarbon receptor signaling (53, 54) (**Figure 1B**). Circulating metabolites, cytokines, and immune-cell-associated signals may then influence blood–brain barrier (BBB) function and the central immune microenvironment (55). Microglial maturation and function are influenced by microbial status (56, 57), while microbial metabolites may modulate neuroinflammatory responses through microglia–astrocyte interactions (58) (**Figure 1C**).

## 3 The intestinal barrier: the entry point for microbial signals into the neuroimmune network

The intestinal epithelium, mucus layer, tight-junction complexes, and mucosal immune system together form a critical interface between the host and luminal microorganisms (59). Under physiological conditions, this barrier permits the selective exchange of nutrients and microbial metabolites while limiting the translocation of microorganisms and potentially harmful microbial products (60). Barrier dysfunction may involve reduced expression, altered localization, or impaired assembly of tight-junction proteins, including occludin, claudins, zonula occludens proteins, and junctional adhesion molecules. It may also arise from goblet-cell dysfunction or depletion, which can reduce mucus production and compromise the protective mucosal layer (61).

Gut dysbiosis, dietary alterations, infection, inflammation, gastrointestinal dysmotility, and medication exposure may all affect intestinal barrier integrity (62, 63). *In vitro* and animal studies indicate that LPS can disrupt epithelial tight-junction organization and increase intestinal permeability (64, 65). In disease settings, impaired barrier function, increased exposure to microbial products, and peripheral inflammatory responses may mutually reinforce one another (65, 66). Nevertheless, in human neurodegenerative diseases, longitudinal studies are needed to determine whether intestinal barrier disruption precedes disease progression, results from it, or reflects shared disease-related factors (67).

Neuroimmune interactions in the gut are also bidirectional (66). Enteric neurons, the vagus nerve, and intestinal immune cells can regulate one another: neuropeptides may modulate immune-cell activation, whereas vagal pathways can influence intestinal macrophage function and inflammatory responses (68–70). Thus, the intestinal barrier should be viewed not as a passive physical structure but as a dynamic interface that integrates microbial, immune, epithelial, and neural signals and may influence the transmission of gut-derived signals to the neuroimmune network.

## 4 Microbiota-derived metabolites and molecular patterns: mediators linking gut immunity and brain immunity

### 4.1 Short-chain fatty acids: context-dependent immunometabolic signals

Short-chain fatty acids (SCFAs), which mainly include acetate, propionate, and butyrate, are important products of dietary fiber fermentation by the gut microbiota (71, 72). SCFAs can participate in host metabolic and immune regulation through G protein-coupled receptor signaling and epigenetic mechanisms (73). Previous studies have shown that SCFAs can promote the secretion of glucagon-like peptide-1 (GLP-1) and peptide YY (PYY) by enteroendocrine cells through free fatty acid receptor 2 (FFAR2) (74, 75), suggesting that they may participate in gut metabolic-neuroendocrine signaling. SCFAs can affect mucosal immune and central immune homeostasis through receptor-dependent signaling, immunometabolic regulation, and epigenetic mechanisms (76). Their directional effects are dependent on the disease model, exposure dose, cell type, and host background.

At the neuroimmune level, microglial maturation and function are associated with host microbial status (77), and SCFAs are considered important candidate mediators that may participate in this process. *In vitro* studies have also shown that SCFAs, either alone or in combination, can modulate selected immune functions of microglia-like cells (78). In an AD animal model, *Clostridium butyricum* intervention was associated with changes in the gut microbiota and butyrate levels, as well as attenuated microglia-mediated neuroinflammation (79, 80). However, these findings do not imply that all SCFAs exert consistent protective effects at all disease stages. Their effects may be influenced by concentration (81), receptor expression (82), host metabolic status, disease model (83), and the immune microenvironment (84, 85).

In PD, intestinal SCFA levels and gut microbial composition differ between patients and age-matched controls (86). This observation supports the possibility that abnormal SCFA metabolism is associated with disease-related alterations in the intestinal ecosystem; however, it does not establish the causal direction of SCFA alterations in PD onset and progression (87, 88).

### 4.2 Bile acids: metabolic nodes in the gut microbiota-liver-immune network

Bile acids are synthesized in the liver, enter the intestinal tract, and can subsequently be transformed by the gut microbiota. Dysbiosis-associated deficiency of secondary bile acids can promote intestinal inflammation (89), suggesting that bile acids are not only molecules involved in lipid absorption but may also influence mucosal immune homeostasis.

In individuals with AD, altered bile acid profiles are associated with cognitive impairment (90), and alterations in hepatic bile acids have also been observed in animal models of AD (25). These findings provide clues for a “gut microbiota-bile acid-neuroimmune” axis; however, current evidence remains insufficient to define specific bile acid alterations as direct pathogenic factors in AD (91). Future studies should simultaneously measure microbial functional genes, fecal and blood bile acid profiles, intestinal barrier status, immune phenotypes, and neuropathological indicators.

### 4.3 Tryptophan derivatives: potential clues to AhR-related immune regulation

Tryptophan can be metabolized by the host and microorganisms into multiple derivatives and is associated with the kynurenine pathway and aryl hydrocarbon receptor (AhR) signaling (92). Previous studies have indicated that microbial metabolites can regulate astrocyte responses through microglia, with AhR-related pathways representing one mechanism of particular interest (93).

In APP/PS1 mice, a high-tryptophan diet was associated with alterations in the gut microbiota, AhR activation, and improvements in cognitive impairment and neuroinflammation (94). This finding suggests that tryptophan-microbiota-AhR signaling may participate in neuroimmune regulation in AD models. However, dietary components, host tryptophan metabolism, and microbiota alterations are difficult to fully disentangle (95). Therefore, further validation using colonization models, supplementation with specific metabolites, and receptor-deficient models is required.

### 4.4 LPS and other microbe-associated molecular patterns: potential bridges from local recognition to systemic inflammation

Lipopolysaccharide (LPS) derived from Gram-negative bacteria can activate innate immune recognition pathways and is widely used in experimental models of induced inflammation. Gram-negative bacterial molecules or LPS signals associated with pathology have been detected in AD brain tissue (96, 97). However, these findings primarily provide associative evidence and cannot independently demonstrate that gut-derived LPS enters the brain and drives AD pathology.

Accordingly, LPS should not be described as a universal “decisive cause” of neurodegenerative diseases (98). A more cautious interpretation is that, in the context of intestinal barrier impairment and systemic inflammation, microbe-associated molecular patterns may represent candidate factors that influence peripheral immunity and the threshold for neuroinflammation (99). Their effects in the brain depend on barrier status, host clearance mechanisms, immune context, and disease stage.

## 5 From intestinal mucosal immunity to systemic immunity: relay functions of innate and adaptive immunity

By influencing antigen-presenting cells, intestinal macrophages, innate lymphoid cells, and lymphocytes, the gut microbiota participates in the balance between immune tolerance and inflammatory responses (100). Intestinal macrophages can acquire tissue-specific functional states under the combined influence of local microbial and neural signals (101), whereas enteric glia-associated regulatory factors can also affect group 3 innate lymphoid cells (ILC3s) and intestinal defense (102). Intestinal innate immune cells constitute a potential peripheral window for assessing central immune status (103). Macrophages, dendritic cells, ILC3s, neutrophils, and intestinal epithelial cells in the intestinal mucosa together form the first immune network responsible for recognizing the gut microbiota and maintaining barrier homeostasis (104). Gabanyi et al. found that intestinal macrophages can integrate microbial metabolic signals and enteric neural signals, thereby adopting tissue-specific reparative or proinflammatory phenotypes (105). Ibiza et al. further demonstrated that enteric glial cells regulate ILC3s and interleukin (IL)-22-mediated mucosal defense through glial cell line-derived neurotrophic factor family ligands (102). Recent studies suggest that peripheral monocyte proportions, M1/M2-like polarization of intestinal macrophages (106), ILC3 activity (107), and cytokines (108) including IL-6, IL-1β, tumor necrosis factor-alpha (TNF-α), IL-17A, IL-22, and IL-23 may be associated with microglial activation and the extent of inflammation in the brain (109, 110). Intestinal barrier disruption, circulating LPS, and elevated peripheral inflammatory cytokines are common in PD (111, 112), AD (113), and multiple system atrophy (114–116). Researchers have also noted that combined assessment of gut microbiota dysbiosis and peripheral inflammatory markers may improve early risk stratification for neurodegenerative diseases (117).

With respect to adaptive immunity, T-cell differentiation and regulatory T-cell (Treg) function represent important links between the gut microbiota and systemic immune tolerance (118). Dietary fatty acids can influence CNS autoimmunity through immune processes in the small intestine (119), supporting the concept that the intestinal immune environment can affect distal neuroimmune responses. Adaptive immunity may be a key relay through which intestinal inflammation is transmitted to brain pathology, although causality remains to be established (120). The gut microbiota and its metabolites can affect the differentiation of T helper 1 (Th1) cells, Th17 cells, T follicular helper (Tfh) cells, Tregs, and B cells through antigen presentation, costimulatory signals, and metabolic reprogramming (121). Among these factors, SCFAs generally facilitate the induction of forkhead box P3-positive (Foxp3^+^) Tregs and the maintenance of immune tolerance, whereas intestinal barrier impairment, pathobiont expansion, and increased proinflammatory metabolites may promote Th17 responses and IL-17A release (122, 123). Increased peripheral Th1/Th17 cells, impaired Treg function, and T-cell infiltration into the brain parenchyma have been reported in patients with PD and in animal models (124). Abnormalities in effector memory T cells (125), B-cell subsets (126), and antibody responses (127) have also been observed in AD. However, whether these changes directly drive alpha-synuclein, amyloid-beta, or tau pathology, or instead represent secondary immune responses to chronic neural injury, cannot be determined on the basis of correlational studies alone.

Loss of mucosal immune tolerance may precede overt neuropathology, but longitudinal evidence in human populations is currently lacking. The “gut-origin” hypothesis has been particularly advanced by PD research: constipation, increased intestinal permeability, abnormal alpha-synuclein deposition in the enteric nervous system, and alterations in microbial composition may occur years before the onset of typical motor symptoms (128). Some animal studies indicate that dysbiosis, intestinal inflammation, or exposure to microbial products can accelerate neuroinflammation (129) and protein misfolding (130). Experiments involving germ-free animals (131), antibiotic interventions (132), and fecal microbiota transplantation (133) also support a modulatory role of the gut microbiota in neurodegenerative disease phenotypes (134). However, the observation that “intestinal alterations precede brain pathology” does not mean that “intestinal alterations cause brain pathology.” Age, diet, sleep, constipation, reduced physical activity (135), and medication use, particularly levodopa and antibiotics, can in turn affect the gut microbiota and mucosal immune status (136, 137). Therefore, fecal microbiota, intestinal barrier markers, mucosal immune cells, blood cytokines, and cerebrospinal fluid biomarkers should be tracked concurrently to determine the temporal sequence among declining immune tolerance, peripheral inflammation, and neuropathology.

Different neurodegenerative diseases may share a framework of “barrier impairment-peripheral inflammation-brain immune dysregulation,” but the specific immune pathways involved are disease-specific. PD, AD, ALS, and Huntington’s disease may all exhibit gut dysbiosis, reduced intestinal barrier integrity, elevated peripheral proinflammatory factors, and aberrant microglial activation (138–141). Accordingly, Toll-like receptor 4 (TLR4)/nuclear factor-kappa B (NF-κB) signaling (142), the NLR family pyrin domain containing 3 (NLRP3) inflammasome (83), the IL-1β/IL-6/TNF-α axis (143), Th17/Treg imbalance (144), and alterations in microbial metabolites may constitute shared immune networks across diseases (142, 145). Nevertheless, the core protein pathologies, genetic susceptibility backgrounds, regional brain vulnerability, and modes of immune-cell involvement differ among diseases. For example, PD places greater emphasis on alpha-synuclein-associated antigen responses, the enteric nervous system, and vagal pathways, whereas AD is closely associated with impaired Aβ clearance (146), the complement system (147), cerebrovascular barriers (148), and apolipoprotein E-related immune regulation (149). Therefore, elevated inflammation across different diseases should not be regarded as reflecting a single mechanism. Future studies should compare shared immune modules and disease-specific immune features using standardized sampling, uniform disease staging, and multi-omics analyses, followed by validation in organoids, germ-free animals, humanized microbiota models, and clinical intervention studies.

## 6 BBB, microglia, and astrocytes: central immune integrators

### 6.1 Gut microbiota and blood-brain barrier function

The blood-brain barrier (BBB) is a critical structure that maintains homeostasis within the central nervous system (CNS) (150). It consists of cerebral microvascular endothelial cells and their tight junctions, pericytes, astrocytic endfeet, the basement membrane, and associated immunoregulatory components (151, 152). The BBB not only restricts the entry of harmful peripheral substances and immune cells into the brain parenchyma, but also participates in nutrient transport, metabolic waste clearance, neurovascular coupling, and the maintenance of central immune homeostasis (153). Braniste et al. found in germ-free and microbiota-colonized mice that microbial status was associated with BBB permeability and the expression of tight junction-related proteins, suggesting that the gut microbiota may participate in the regulation of BBB function (154). This study provided important experimental evidence that the gut microbiota can influence central barrier properties. Subsequently, studies have further suggested that gut dysbiosis and associated alterations in microbial metabolites may contribute to BBB dysfunction in neurodegenerative diseases, such as Alzheimer’s disease (AD) and Parkinson’s disease (PD), by regulating endothelial inflammatory responses (155), tight junction proteins (156), oxidative stress (157), and the perivascular immune microenvironment (158).

### 6.2 Microglia: key targets through which microbial signals maintain central immune homeostasis

Microglia are the principal resident myeloid immune cells in the CNS and play multiple roles in brain development (159), synaptic pruning (160), myelin maintenance (161), host defense (162), cellular debris clearance (163), and the regulation of neuroinflammation (164). Using germ-free animals and microbial colonization models, Daniel et al. demonstrated that the host microbiota continuously influences microglial maturation, morphology, transcriptional features, and immune responsiveness (165, 166). Gut-derived signals can affect microglia through multiple mechanisms, including circulating metabolites, peripheral inflammatory mediators, vagus nerve-associated pathways, and alterations in BBB function (167, 168). Among these signals, SCFAs, tryptophan metabolites, bile acid derivatives, and lipid metabolites are considered important candidate mediators (153, 169).

In AD models, antibiotic-induced perturbation of the gut microbiota has been associated with neuroinflammatory responses (170), amyloid deposition (171), and changes in microglial function. Tianwang Buxin Pill improves cognitive function in APP/PS1 mice by attenuating neuronal damage and modulating synaptic plasticity. In addition, germ-free APP/PS1 mice exhibit reduced Aβ amyloid pathology, while microbiota reconstitution can alter their pathological phenotype (172). These studies suggest that the gut microbiota may participate in AD-associated neuroinflammation and protein aggregation. In PD models, it was found that the gut microbiota can regulate motor dysfunction (173), microglial activation (174), and alpha-synuclein-associated pathological phenotypes (175), providing relatively strong support from animal studies for microbiota involvement in PD progression. However, current evidence remains influenced by animal sex, age, genetic background, antibiotic regimens, model type, dietary conditions, and microbiota reconstitution status. Whether alterations in the gut microbiota are causal drivers of microglial abnormalities or secondary consequences of neurodegeneration and treatment exposure requires further validation through prospective population studies and mechanistic experiments.

### 6.3 Microglia-astrocyte interactions: central amplification or constraint of metabolite responses

Microglia and astrocytes together constitute a core network for innate immune responses and tissue homeostasis in the brain (176). Their bidirectional communication may represent an important relay through which gut microbiota-derived metabolic signals are translated into neuroinflammation and neurodegenerative pathology (177). Microglia can sense LPS (178), SCFAs (179), tryptophan metabolites (180), bile acid derivatives (181), and peripheral inflammatory factors (182), and regulate phagocytosis (LAP, LANDO) (183, 184), chemotaxis (185), antigen presentation (186), and cytokine secretion through pathways including TLR4/NF-κB signaling, the NLRP3 inflammasome, AhR signaling (187), and type I interferon (IFN-I) pathways (188). Activated or reprogrammed microglia can further release signaling molecules, including interleukin (IL)-1α (189), TNF-α, complement component 1q (C1q) (190), IL-6, and transforming growth factor-beta (TGF-β) (191), thereby inducing or reshaping astrocyte reactive states (192, 193). Microbiota-derived tryptophan metabolites can regulate the expression of TGF-α and vascular endothelial growth factor B (VEGF-B) by activating AhR in microglia (194, 195), thereby influencing astrocyte-mediated inflammatory responses. These study provided relatively direct experimental evidence for a regulatory axis linking “gut microbiota-derived metabolites-microglia-astrocytes.”

It was proposed that microglia-derived IL-1α, TNF, and C1q can induce A1-like astrocytes with neurotoxic features (196). At the same time, a growing body of research indicates that the conventional binary classification of astrocytes as “A1/A2” and microglia as “M1/M2” does not adequately reflect the true heterogeneity of glial cells under disease conditions (197). Single-cell and spatial omics studies have shown that glial cells display dynamic transcriptional states that are brain region-specific, disease stage-dependent, and microenvironment-dependent in PD (198), AD (198), and ALS (199). Their functions may promote abnormal protein aggregation (200), oxidative stress (201), synaptic loss (201), neuronal injury (202), and BBB disruption (203), but may also contribute to cellular debris clearance (204), neurotrophic support (205), maintenance of metabolic homeostasis (164), and tissue repair (206). Therefore, microglia-astrocyte interactions are not a unidirectional proinflammatory process; rather, they may determine whether central inflammatory responses are persistently amplified or effectively constrained (**Figure 1D**).

## 7 Evidence for the gut-immune-brain axis in neurodegenerative diseases

### 7.1 Parkinson’s disease: gut abnormalities, alpha-synuclein pathology, and neuroinflammation

Parkinson’s disease (PD) is a neurodegenerative disease in which evidence for the gut-brain axis has accumulated relatively extensively (207). Multiple clinical studies have found alterations in gut microbial composition, microbial metabolic function, and SCFA levels in patients with PD (208, 209). In addition, gastrointestinal symptoms, including constipation and defecation difficulties, often precede the onset of typical motor symptoms, and some patients also exhibit intestinal mucosal inflammation and impaired intestinal barrier function (210, 211). These findings suggest that gut microbial dysbiosis, local immune abnormalities, and altered enteric nervous system function may participate in the early pathological processes of PD (212). However, delayed gastrointestinal motility, changes in dietary patterns, the use of antiparkinsonian medications, and disease progression itself can in turn reshape the gut microbiota (213). Therefore, clinically observed microbial differences may still be affected by multiple confounding factors.

Animal studies have provided more direct causal clues regarding the involvement of the gut microbiota in PD pathology (214). In an alpha-synuclein-related PD model, gut microbiota can regulate motor dysfunction (215), and microglial activation (216), suggesting that the microbiota may participate in PD-related neurodegenerative processes. Wei et al. further proposed that pathological alpha-synuclein may propagate along gut-brain-associated neural pathways and contribute to central pathological changes (217, 218). Overall, current evidence supports a close relationship among the gut microbiota, intestinal mucosal immunity, and alpha-synuclein pathology. However, the temporal sequence, modes of interaction, and interindividual variability of gut-derived alpha-synuclein abnormalities, microbial alterations, immune activation, and central nervous system pathology in patients remain incompletely understood. Future studies should prioritize multi-time-point longitudinal investigations in prodromal PD cohorts, individuals with rapid eye movement sleep behavior disorder, and genetically high-risk populations to determine the actual position of gut abnormalities in the course of PD (**Figure 2A**).

**Figure 2:**
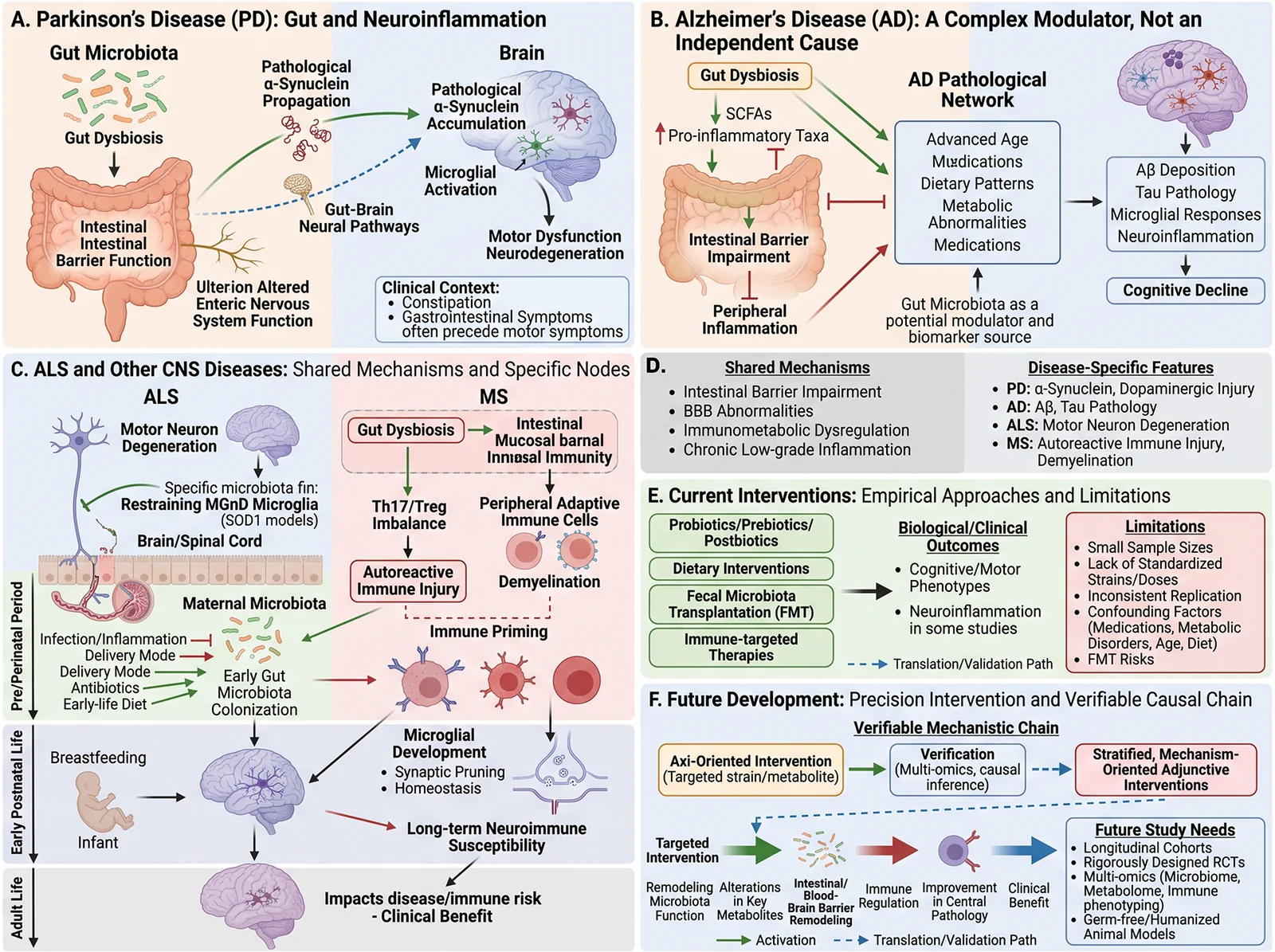
Gut microbiota–immune–brain interactions in neurological disease. **(A)** In PD, dysbiosis may impair the intestinal barrier and enteric nervous system, facilitating α-synuclein propagation, microglial activation, and neurodegeneration. **(B)** In AD, dysbiosis and altered SCFAs may promote barrier dysfunction, inflammation, amyloid-β/tau pathology, and cognitive decline. **(C)** Perinatal factors may shape microbiota, microglia, and neuroimmune susceptibility; disease-specific mechanisms include MGnD modulation in ALS and Th17/Treg imbalance with demyelination in MS. **(D)** Shared and disease-specific mechanisms are summarized. **(E)** Candidate interventions and current limitations are outlined. **(F)** Longitudinal multi-omics, clinical trials, and experimental models may validate causal pathways and guide precision therapies. Arrows indicate proposed relationships; dashed arrows denote translational steps requiring validation.

### 7.2 Alzheimer’s disease: gut dysbiosis may participate in the pathological network but is not an independent cause

Alterations in gut microbial composition and microbial metabolite profiles have been reported in patients with Alzheimer’s disease (AD) and related animal models (219). Some studies have found an increased abundance of proinflammation-associated microbial taxa and a reduced abundance of taxa capable of producing SCFAs in patients with AD (220). These changes may be associated with peripheral inflammation, impaired intestinal barrier function, amyloid deposition, and cognitive decline. In transgenic animal models, including APP/PS1 mice, microbiota remodeling, antibiotic treatment, or interventions with specific probiotics may be accompanied by changes in Aβ deposition, neuroinflammation, microglial responses, cognitive phenotypes, and levels of gut-derived metabolites (221–223). These studies suggest that the gut microbiota may participate in the pathological network of AD through immune-, metabolic-, and barrier-related mechanisms.

Current studies in individuals with AD are predominantly cross-sectional and are characterized by substantial heterogeneity in sample size, sequencing platforms, disease staging, and statistical adjustment strategies. Advanced age, dietary patterns, reduced physical activity, declining nutritional status, abnormalities in glucose and lipid metabolism, antibiotic exposure, and other long-term medications may independently or jointly influence both the gut microbiota and AD-related clinical phenotypes (224). Therefore, the gut microbiota should currently be regarded as a potential modulator within the pathological network of AD, a possible source of patient-stratification biomarkers, and a potential target for therapeutic intervention, rather than as an independently established cause (140).

Future studies should rigorously control for potential confounders and integrate longitudinal cohorts with cerebrospinal fluid Aβ and tau biomarkers, neuroimaging measures, and microbial functional omics. Such approaches will be essential for testing the proposed sequence linking altered microbial functions, metabolite disturbances, immune dysregulation, and AD progression (**Figure 2B**). Although associations between gut microbial features and AD-related cognitive or pathological measures have been reported, their reproducibility across cohorts remains variable. Experimental studies of selected microbial metabolites provide biologically plausible mechanistic hypotheses; however, direct evidence that these metabolites modify disease progression in patients remains limited.

### 7.3 ALS, MS, and other central nervous system diseases: coexisting shared immune nodes and disease-specific mechanisms

Alterations in the gut microbiota have also been reported in patients with amyotrophic lateral sclerosis (ALS) and in ALS animal models (225). Recent clinical studies suggest that dysfunction of gastrointestinal tract may play a role in ALS, Letizia et al. proposed that *Enterobacteria*, *Bifidobacterium* spp., *Lactobacillus* spp., *Clostridium sensu stricto*, *Escherichia coli* and yeast may indicate that a real dysbiosis status is not evident in the ALS patients (226). Laura et al. found that the microbiota play a protective role in the SOD1 model of ALS by restraining MGnD microglia, which is opposite to other neurologic disease models, and sheds new light on the importance of disease-specific interactions between microbiota and microglia (226). These findings provide preliminary support for the involvement of the gut microbiota in ALS pathological processes. However, this field continues to face limitations, including small cohort sizes, insufficient sample reproducibility, substantial disease heterogeneity, and inadequate mechanistic validation. In particular, patients with ALS commonly experience dysphagia, altered nutritional status, restricted activity, and respiratory support treatment (227). These factors may all influence the gut microbiota and increase the difficulty of causal inference.

Although multiple sclerosis (MS) is primarily considered a disease characterized by autoimmune demyelination within the central nervous system (228) (**Figure 2C**), the association among gut dysbiosis (229), Th17/Treg imbalance (230), and abnormal mucosal immunity provides an important reference for understanding gut-immune-CNS interactions (231). Compared with PD, AD, and ALS, evidence for the involvement of peripheral adaptive immune cells in central pathology is relatively more established in MS (232) (**Figure 2D**). Although different diseases may share mechanistic nodes, including intestinal barrier impairment, BBB abnormalities, immunometabolic dysregulation, microglial responses, and chronic low-grade inflammation, they should not be simply classified into a single pathological model (233). PD primarily involves alpha-synuclein pathology and dopaminergic neuronal injury; AD is characterized by Aβ and tau pathology; ALS is distinguished by motor neuron degeneration; and MS is mainly characterized by autoreactive immune injury and demyelination (234, 235). Therefore, future research should identify disease-specific microbial functions, metabolite signals, and immune pathways within a shared mechanistic framework.

## 8 Perinatal microbiota and immune priming: a developmental window for neuroimmune susceptibility

The perinatal period is a critical stage during which host microbial colonization, maturation of the innate and adaptive immune systems, and neurodevelopment occur concurrently. It was demonstrated that the maternal microbiota can shape postnatal immune responsiveness by influencing early immune development in the fetus or offspring (236). López-Moreno et al. also noted that perinatal microbial exposure, maternal immune status, gut microbiota colonization, and neurodevelopment are closely and dynamically interconnected (237). Meanwhile, microglia undergo stage-specific programmed changes during the embryonic period, perinatal period, and early postnatal life, and participate in synaptic pruning, neural circuit formation, and the establishment of brain homeostasis (238, 239) (**Figure 2B**). Therefore, factors including maternal microbial composition, infection or inflammation during pregnancy, mode of delivery, antibiotic exposure, breastfeeding, and early-life diet may theoretically alter long-term neuroimmune susceptibility in offspring by affecting immune priming and microglial development (240).

Nevertheless, the association between perinatal microbiota abnormalities and the risk of neurological disorders in offspring remains exploratory, and findings from animal studies should not be directly extrapolated to human disease outcomes (241). Maternal microbiota perturbation or immune activation observed in animal models cannot directly demonstrate that such changes determine the occurrence of a specific neurodevelopmental or neurodegenerative disease in offspring. Human diseases are typically influenced by multiple factors, including genetic background, family environment, socioeconomic factors, dietary patterns, exposure to infection, and health status across the life course (242). Future studies should establish mother-infant longitudinal cohorts with adequate sample sizes and long-term follow-up. Maternal, placental, cord blood, infant fecal, and peripheral blood samples should be collected in parallel, and microbiome, metabolome, immune phenotyping, brain imaging, and neurobehavioral developmental measures should be integrated to systematically assess the long-term effects of perinatal microbiota and immune priming on subsequent neurological health.

## 9 Therapeutics and translation: from broad “microbiota modulation” to verifiable mechanism-targeted interventions

Probiotics, prebiotics, postbiotics, dietary interventions, fecal microbiota transplantation (FMT), and immune-targeted therapies are all considered potential intervention strategies for modulating the gut-immune-brain axis (**Figure 2E**). Some animal studies have shown that probiotic supplementation, reconstitution with specific microbial communities, or dietary modulation may be accompanied by improvements in cognitive, motor, and neuroinflammatory phenotypes (243, 244). For example, *Lactobacillus plantarum* combined with memantine improved cognitive impairment in APP/PS1 mice and altered gut microbial composition and inflammation-related indicators (245). In clinical research, a small randomized controlled trial conducted by Nagham et al. suggested that probiotic supplementation may be associated with improvements in selected cognitive and metabolic measures in patients with AD (246). These findings provide preliminary support for the clinical translation of microbiota-based interventions; however, current evidence remains insufficient to support their use as standard treatments for neurodegenerative diseases.

Several limitations continue to hinder clinical translation. Existing studies are often small and differ substantially in microbial strains, doses, formulations, treatment duration, and outcome measures, limiting cross-study comparability and reproducibility. In addition, demographic and population-related factors—including age, sex, ancestry, geographical location, diet, socioeconomic environment, medication exposure, comorbidities, and disease stage—can influence both the gut microbiota and disease phenotypes (247). Technical differences in sample collection, sequencing, metabolite measurement, and bioinformatic processing may further contribute to inconsistent findings. Most studies have also not established a continuous causal relationship linking intervention-induced microbial or functional changes to metabolite production, immune remodeling, and clinical benefit. For FMT, donor selection, pathogen transmission, long-term safety, recipient compatibility, and procedural standardization require further evaluation. These limitations support a shift from broad, empirical “microbiota modulation” toward precision interventions with defined mechanisms, measurable targets, and stratifiable therapeutic responses.

Future studies should use harmonized protocols and multi-time-point, multilevel designs to assess the temporal relationships among intervention exposure, microbial functions, key metabolites, intestinal and systemic immune phenotypes, BBB integrity, glial states, neuropathology, and clinical outcomes. Microbiota-associated biomarkers should be evaluated in independent, multicenter, longitudinal cohorts using prespecified analytical pipelines. Because single bacterial taxa may vary across populations, robust biomarkers may instead comprise reproducible microbial functions, metabolic pathways, or integrated host–microbe signatures. Mechanistic studies may combine germ-free or humanized-microbiota models, defined-strain colonization, fecal microbiota transfer, candidate-metabolite supplementation or rescue, and blockade of relevant receptors or signaling pathways to test the necessity and sufficiency of candidate microbial mechanisms. Clinical trials should include prespecified microbiological, metabolomic, immunological, barrier-function, and neurobiological endpoints, together with longitudinal multi-omics profiling and appropriate causal analyses.

Establishing a reproducible mechanistic chain linking intervention, microbial functional change, metabolite production, immune and barrier remodeling, central pathology, and clinical benefit will be essential for advancing gut microbiota-based therapies from exploratory approaches to stratified, mechanism-oriented adjunctive interventions for neurodegenerative diseases (**Figure 2F**).
